# Incidence of Arrhythmias and Their Prognostic Value in Patients With Multiple Myeloma

**DOI:** 10.3389/fcvm.2021.753918

**Published:** 2021-11-15

**Authors:** Yongxin Li, Manyun Tang, Liang Zhong, Suhua Wei, Jingzhuo Song, Hui Liu, Chaofeng Sun, Jie Wang

**Affiliations:** ^1^Department of Cardiovascular Surgery, The First Affiliated Hospital of Xi'an Jiaotong University, Xi'an, China; ^2^Department of Cardiovascular Medicine, The First Affiliated Hospital of Xi'an Jiaotong University, Xi'an, China; ^3^Department of Hematology, The First Affiliated Hospital of Xi'an Jiaotong University, Xi'an, China; ^4^Department of Medical Oncology, The First Affiliated Hospital of Xi'an Jiaotong University, Xi'an, China; ^5^Biobank, The First Affiliated Hospital of Xi'an Jiaotong University, Xi'an, China

**Keywords:** risk stratification, arrhythmias, multiple myeloma, prognosis, cardiovascular complication

## Abstract

**Background:** Arrhythmias are common cardiovascular complications in multiple myeloma (MM) patients and are related to a poor prognosis.

**Objective:** This study aimed to assess the burden of arrhythmias and their prognostic value in patients with MM.

**Methods:** This was a retrospective study of patients with MM between January 2015 and April 2020 at the First Affiliated Hospital of Xi'an Jiaotong University. The incidence of arrhythmia and associated risk factors were evaluated. The relationship between the type of arrhythmia and survival was analyzed.

**Results:** A total of 319 patients with MM were identified, and 48.0% (153/319) had arrhythmias. The most common type of arrhythmia was sinus tachycardia (ST) (15.0%, 48/319), followed by sinus bradycardia (SB) (14.4%, 46/319), premature atrial contractions (PACs) (6.3%, 20/319), conduction disorders (CDs) (6.0%, 19/319), atrial fibrillation (AF) (6.0%, 19/319), premature ventricular contractions (PVCs) (4.4%, 14/319) and paroxysmal supraventricular tachycardia (PSVT) (0.6%, 2/319). The patients with arrhythmias had higher levels of log NT-proBNP and creatinine, greater bortezomib use, and a higher incidence of diabetes than those without arrhythmias (*P* < 0.05). The all-cause mortality rates of patients without arrhythmias and those with AF, ST, PACs, CDs, SB, and PVCs were 50.6% (84/166), 73.7% (14/19), 60.4% (29/48), 60.0% (12/20), 52.6% (10/19), 34.8% (16/46), and 28.6% (4/14), respectively. In a subgroup analysis of patients experiencing different types of arrhythmias, patients with SB had lower all-cause mortality than patients with AF (*P* < 0.01). Univariate and multivariate Cox analyses showed that there was a positive statistically significant association between SB and survival (HR: 0.592 [0.352–0.998], *P* = 0.049) in a subgroup analysis of different arrhythmias.

**Conclusions:** Patients with MM had a heavy arrhythmia burden, and in this study, approximately half of MM patients had arrhythmias. MM patients with SB were associated with lower all-cause mortality than those with AF. SB might be an independent positive factor for prognosis.

## Introduction

Multiple myeloma (MM) is one of the most common malignant hematologic tumors, accounting for 10% of hematopoietic tissue tumors and 1% of all cancers (1). MM is a clonal plasma-cell neoplasm, and its features include hypercalcemia, renal dysfunction, anemia, bone lesions, and other organ damage (2). With advancements in biologically targeted treatment in recent years, the survival of MM patients has improved significantly (3).

Cardiac complications such as arrhythmias are common in patients with MM (4, 5). One study including ~20% of all United States community hospitals used a nationwide inpatient sample dataset and reported the burden of cardiac arrhythmias in patients with MM (6). The percentage of patients with arrhythmias among MM patients (20.0%, 18,064/88,507) was found to be greater than that among the general population (13.8%). Many factors have been reported to contribute to the increased incidence of cardiac arrhythmias in patients with MM, such as older age, comorbid cardiovascular conditions, chemotherapeutic agents, and the treatment of coexisting cardiac disease, cardiac amyloidosis, autologous stem cell transplantation (ASCT), and electrolyte abnormalities (6–11). In our previous work, the QTc interval and heart rate were independently associated with all-cause mortality in patients with MM (12, 13). However, the burden of different arrhythmias and the relation between these arrhythmias and prognostic survival in MM patients remain unclear in real-world studies. Therefore, this study aimed to retrospectively investigate the prevalence of different arrhythmias and evaluate their prognostic potential to guide the management of these patients.

## Methods

### Study Population

**Figure 1:**
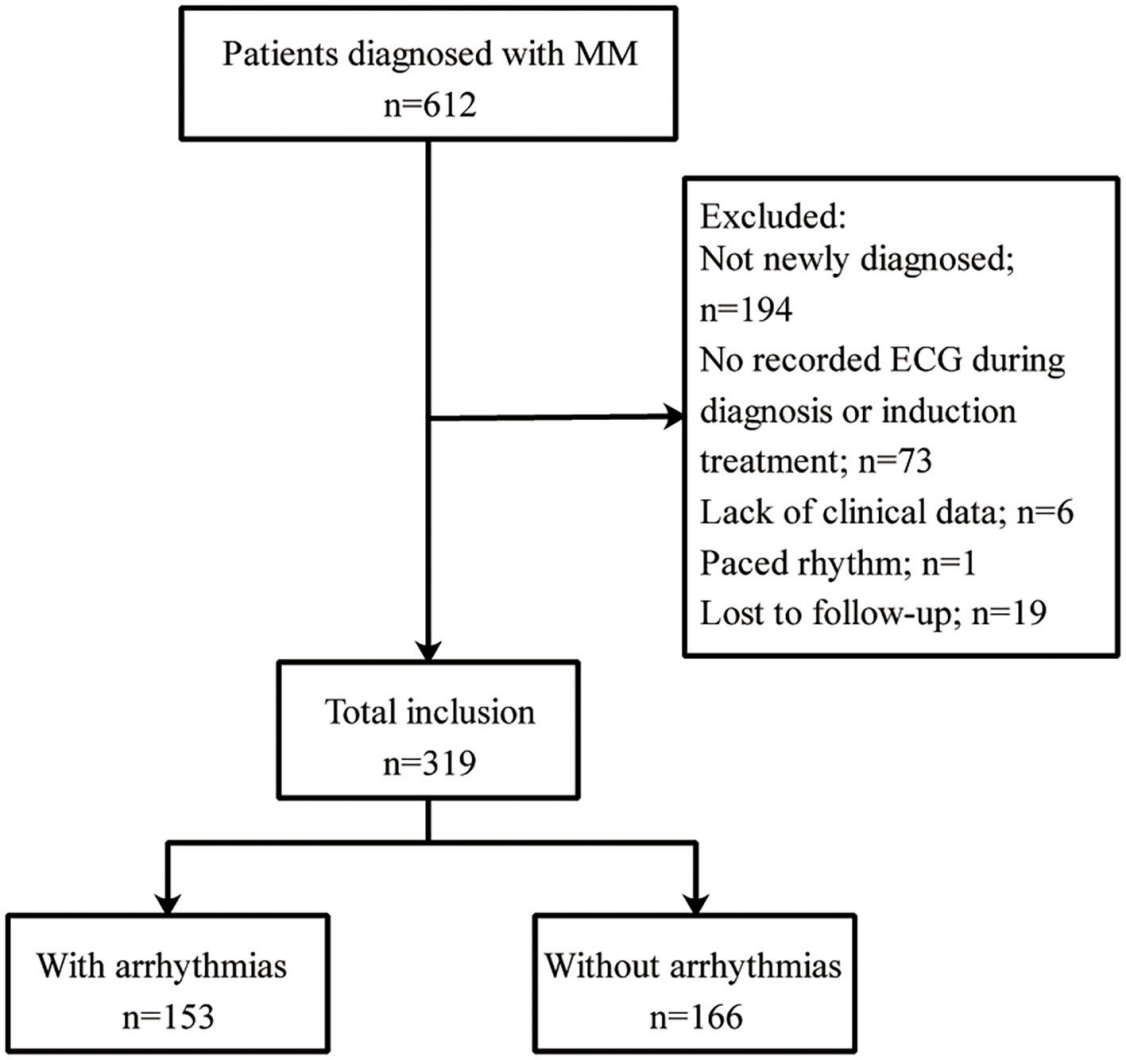
Patient selection flowchart. MM, multiple myeloma; ECG, electrocardiogram.

This retrospective study was approved by the Ethics Committee of the First Affiliated Hospital of Xi'an Jiaotong University (Approval No. XJTU1AF2020LSK-179) and was conducted in accordance with the guidelines of the Declaration of Helsinki. Informed consent was obtained from all participants. Patients diagnosed with MM and hospitalized between January 2015 and April 2020 at the First Affiliated Hospital of Xi'an Jiaotong University were included. The exclusion criteria were as follows: (1) age younger than 18 years; (2) patients who were not newly diagnosed; (3) patients with missing data on electrocardiograms (ECGs) during diagnosis or induction treatment; (4) lack of clinical data; (5) patients with a paced rhythm; and (6) patients who were lost to follow-up. The flowchart of the study population is shown in Figure 1. Thus, 319 patients were ultimately enrolled in the analysis.

### Data Collection

All anonymized data including clinical information, personal history, and laboratory examination results were collected from the electronic medical record system of the hospital and analyzed. The diagnosis of MM was confirmed by a hematologist. Induction therapy was identified according to the European Society for Medical Oncology (ESMO) Clinical Practice Guidelines and National Comprehensive Cancer Network (NCCN) guidelines (14, 15). The International Staging System (ISS) was used for patient stratification (16). Pulmonary hypertension (PH) was defined as an estimated right ventricular systolic pressure >40 mm Hg on transthoracic echocardiogram (17).

Arrhythmias include ventricular and supraventricular arrhythmias which are defined by American College of Cardiology/American Heart Association/ Heart Rhythm Society (ACC/AHA/HRS) Guideline (18–20). A 12-lead ECG was used to detect arrhythmias in the study. The type of heart rhythm from ECGs was extracted, including atrial fibrillation (AF), atrial flutter, paroxysmal supraventricular tachycardia (PSVT), premature atrial contractions (PACs), premature ventricular contractions (PVCs), sinus tachycardia (ST), sinus bradycardia (SB) and conduction disorders (CDs) (right bundle branch block, left bundle branch block and atrioventricular block) and normal sinus rhythm (without arrhythmias). Patients were categorized into the group with arrhythmias and the group without arrhythmias according to the ECG results.

### Outcome Variables

The study endpoint was all-cause mortality. All participants were followed up until death or November 2020. Follow-up measures included outpatient visits, telephone calls or other electronic communications due to social quarantine measures during the coronavirus 2019 (COVID-19) pandemic.

### Statistical Analysis

All statistical analyses were conducted using SPSS version 24. All continuous variables were presented as the means ± standard deviations or medians (interquartile ranges) and were compared using Student's *t*-test. The Kruskal-Wallis test was used when the data were not normally distributed. Categorical variables were expressed as percentages and compared using Pearson's chi-square test or Fisher's exact test to explore differences between groups in terms of all-cause mortality. Univariable logistic regression models were used to determine the risk of arrhythmia. Survival curves were plotted by the Kaplan-Meier method to depict differences in survival, and the log-rank test was utilized to assess statistical significance. Univariate and multivariate Cox proportional hazards models were used to identify independent predictors for the type of arrhythmia. The hazards were proportional. A *P* < 0.05 was considered statistically significant.

## Results

### Baseline Clinical Characteristics

A total of 319 patients (mean age: 61.7 ± 9.8 years) were included, and 60.2% (192/319) were male. Among these patients, 153 patients (48.0%) had arrhythmias (Figure 1). There were no significant differences between the groups in terms of age, sex or laboratory findings, but differences in log N-terminal pro-brain natriuretic peptide (NT-proBNP) and creatinine levels, bortezomib use, supportive care measures and diabetes incidence were observed (Table 1). The patients with arrhythmias had higher levels of log NT-proBNP (3.08 ± 0.80 vs. 2.89 ± 0.78 pg/mL, *P* = 0.028) and creatinine [1.01 (0.70–2.53) vs. 0.85 (0.65–1.54) mg/dL, *P* = 0.038]. Chemotherapy agents including bortezomib, lenalidomide, thalidomide, anthracyclines, cyclophosphamide and supportive care measures were compared between the two groups. The proportion of patients who received bortezomib (54.2 vs. 40.4%, *P* = 0.013) was higher in patients with arrhythmias than in those without arrhythmias. However, the group with arrhythmias had a lower rate of receiving supportive care measures (6.5 vs. 13.3%, *P* = 0.046). For comorbidities, the percentage of patients with diabetes was higher in the group with arrhythmias than in the group without arrhythmias (16.3 vs. 8.4%, *P* = 0.031). Logistic regression analysis demonstrated that the log NT-proBNP level, bortezomib use and diabetes were independent predictors of arrhythmias (Supplementary Table 1). In terms of the transthoracic echocardiographic parameters, left ventricular ejection fraction, left ventricular end-systolic diameter, left ventricular ejection fraction and PH were evaluated. No significant difference was found between the two groups.

**Table 1:** Baseline characteristics of all patients.

**Patient demographics**	**Total**	**Arrhythmias**		***P***
		**With**	**Without**	
	***N* = 319**	***N* = 153**	***N* = 166**	**value**
Age (years)	61.7 ± 9.8	62.2 ± 9.9	61.3 ± 9.6	0.389
Male, *n* (%)	192 (60.2)	98 (64.1)	94 (56.6)	0.176
Log NT-proBNP (pg/mL)	2.98 ± 0.80	3.08 ± 0.80	2.89 ± 0.78	0.028
LDH (U/L)	224.77 ± 153.35	239.73 ± 198.05	210.98 ± 93.76	0.094
Serum albumin (g/L)	32.35 ± 6.40	32.58 ± 6.57	32.14 ± 6.24	0.548
BUN (mmol/L)	6.66 (5.17–10.31)	6.87 (5.26–10.68)	6.43 (4.99–9.85)	0.188
Creatinine (mg/dL)	0.92 (0.67–2.13)	1.01 (0.70–2.53)	0.85 (0.65–1.54)	0.038
eGFR (mL/min/1.73 m^2^)	77.0 (29.1–119.9)	71.3 (26.1–111.9)	82.2 (40.3–127.4)	0.051
Serum calcium (mmol/L)	2.27 ± 0.38	2.29 ± 0.40	2.25 ± 0.35	0.314
Serum potassium (mmol/L)	3.94 ± 0.60	3.94 ± 0.56	3.94 ± 0.63	0.980
Serum sodium (mmol/L)	139.4 ± 4.7	139.4 ± 4.8	139.4 ± 4.7	0.926
**Complete blood count**				
White blood cell (×10^9^/L)	5.34 ± 2.45	5.16 ± 2.20	5.51 ± 2.65	0.210
Hemoglobin (g/L)	85.2 ± 22.2	84.7 ± 23.2	85.7 ± 21.4	0.688
Platelet (×10^9^/L)	164.4 ± 80.6	162.0 ± 88.4	166.6 ± 72.9	0.610
**Immune type of myeloma**, ***n*** **(%)**				
IgG/IgA/IgD	240 (75.2)	114 (74.5)	126 (75.9)	0.650
Light chain κ/λ	73 (22.9)	35 (22.9)	38 (22.9)	
None secreted	6 (1.9)	4 (2.6)	2 (1.2)	
**ISS stage**, ***n*** **(%)**				
I	45 (14.1)	20 (13.1)	25 (15.1)	0.813
II	145 (45.5)	72 (47.0)	73 (44.0)	
III	129 (40.4)	61 (39.9)	68 (40.9)	
Treatment^*^, ***n*** **(%)**				
Bortezomib	150 (47.0)	83 (54.2)	67 (40.4)	0.013
Lenalidomide	30 (9.4)	16 (10.5)	14 (8.4)	0.536
bortezomib and lenalidomide	21 (6.6)	13 (8.5)	8 (4.8)	0.186
Thalidomide	71 (22.3)	36 (23.5)	35 (21.1)	0.600
Anthracyclines^#^	134 (42.0)	68 (44.4)	66 (39.8)	0.397
Liposomal daunorubicin	45 (14.1)	26 (17.0)	19 (11.4)	0.155
Cyclophosphamide^$^	135 (42.3)	66 (43.1)	69 (41.6)	0.777
Supportive care	32 (10.0)	10 (6.5)	22 (13.3)	0.046
**Comorbidities**, ***n*** **(%)**				
CAD	19 (6.0)	12 (7.8)	7 (4.2)	0.172
Hypertension	96 (30.1)	53 (34.6)	43 (25.9)	0.089
Diabetes	39 (12.2)	25 (16.3)	14 (8.4)	0.031
**Echocardiographic parameters**, ***n*** **= 199**				
LVEDD, mm	50.1 ± 5.1	50.6 ± 4.7	49.6 ± 5.5	0.175
LVESD, mm	31.3 ± 4.5	31.8 ± 4.8	30.7 ± 4.1	0.077
IVS, mm	8.5 ± 1.3	8.4 ± 1.2	8.6 ± 1.4	0.557
LVEF (%)	66.7 ± 6.5	66.2 ± 7.0	67.3 ± 5.9	0.228
PH, *n* (%)	20 (10.1)	12 (12.1)	8 (8.0)	0.334

*Median (interquartile range), mean ± SD; NT-proBNP, N-terminal pro-brain natriuretic peptide; LDH, lactate dehydrogenase; eGFR, estimated glomerular filtration rate; ISS, International Staging System; CAD, coronary artery disease; LVEDD, left ventricular end-diastolic diameter; LVESD, left ventricular end-systolic diameter; IVS, interventricular septum; LVEF, left ventricular ejection fraction; PH, pulmonary hypertension*.

* *The patients had taken the agents*.

# *Anthracyclines included epirubicin and pirubicin*.

$ *Included cyclophosphamide and ifosfamide*.

### Characteristics and Incidence of Arrhythmias

Overall, 153 (48.0%) of 319 patients had arrhythmias. The types of abnormal rhythms observed included AF, PSVT, PACs, PVCs, ST, SB, and CDs (right bundle branch block, left bundle branch block and first-degree and Mobitz I atrioventricular block). The most common arrhythmia was ST (15.0%, 48/319), followed by SB (14.4%, 46/319), PACs (6.3%, 20/319), CDs (6.0%, 19/319), AF (6.0%, 19/319), PVCs (4.4%, 14/319), and PSVT (0.6%, 2/319) (Table 2). There was only one patient with atrial flutter, and this patient was included in the AF group. In addition, there were 15 patients with more than one type of arrhythmia (Supplementary Table 2). Among these patients, it is important to note that there was one patient who had three types of arrhythmias: ST and right and left bundle branch block. The all-cause mortality was 40.0% (6/15) among patients with more than one type of arrhythmia. There was no significant difference in prognosis between the patients with one arrhythmia and those with more than one arrhythmia (*P* > 0.05).

**Table 2:** Characteristics of arrhythmias in MM patients.

**Type of arrhythmia**	**Number of patients**	**Incidence among the MM cohort (%)**	**Distribution of arrhythmias (%)**
AF^&^	19	6.0	12.4
PSVT	2	0.6	1.3
PBs^$^	33	10.3	21.6
PACs	20	6.3	13.1
PVCs	14	4.4	9.2
ST	48	15.0	31.4
SB	46	14.4	30.1
CDs^%^	19	6.0	12.4
RBBB	10	3.1	6.5
LBBB	3	0.9	2.0
First-degree and	7	2.2	4.6
Mobitz I AV block			
Total^@^	153	48.0	

*MM, multiple myeloma; AF, atrial fibrillation; PSVT, paroxysmal supraventricular tachycardia; PBs, premature beats; PACs, premature atrial contractions; PVCs, premature ventricular contractions; ST, sinus tachycardia; SB, sinus bradycardia; CDs, conduction disorders; RBBB, right bundle branch block; LBBB, left bundle branch block; AV, atrioventricular*.

& *There was one patient with atrial flutter included in the atrial fibrillation group*.

$ *There was one patient with both premature atrial and ventricular contractions*.

% *There was one patient with both right and left bundle branch block*.

@ *Fifteen patients had more than one type of arrhythmia*.

### Prognostic Value of Arrhythmias

**Figure 2:**
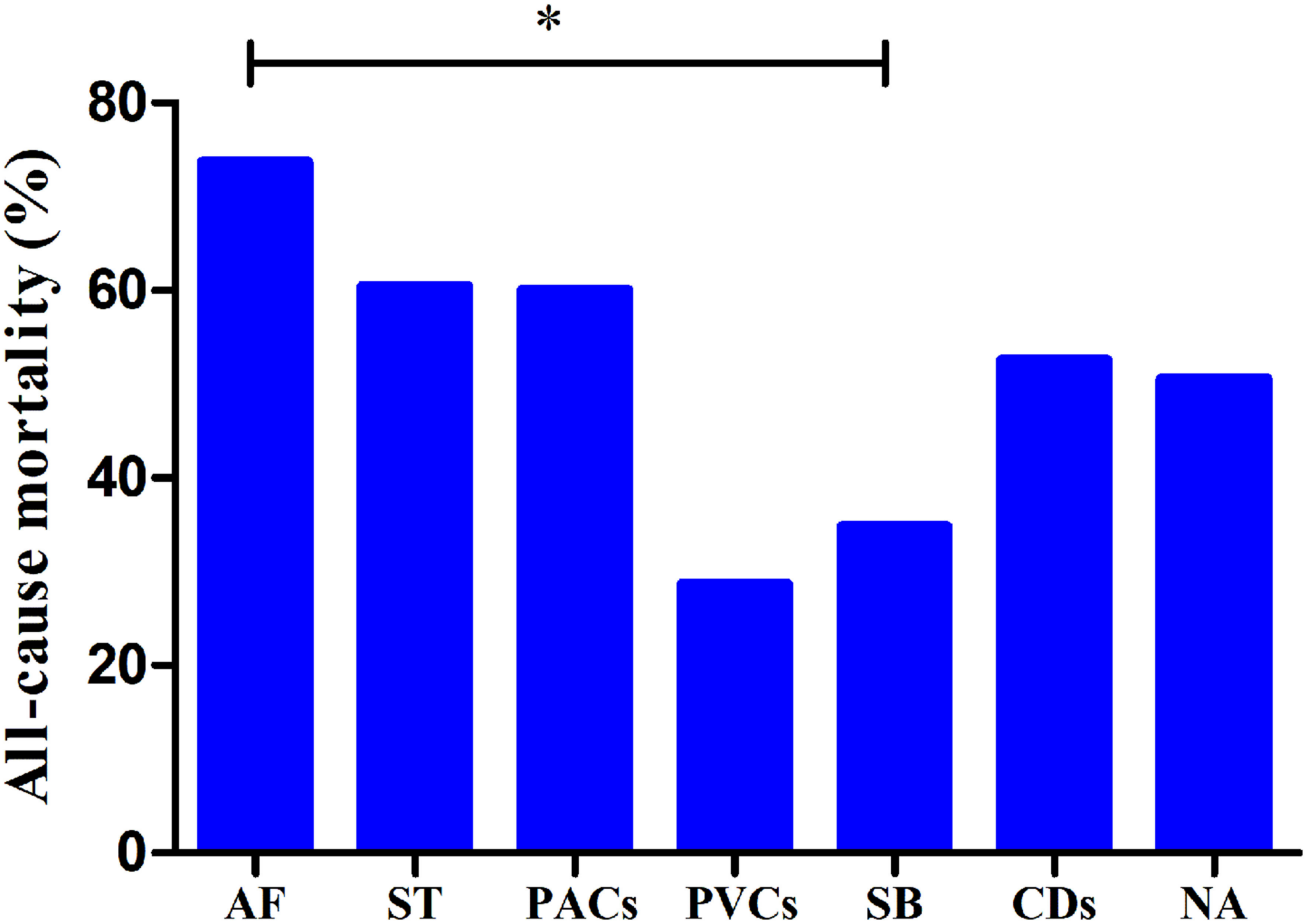
All-cause mortality rates in patients with different types of arrhythmias. *P < 0.01; AF, atrial fibrillation; ST, sinus tachycardia; PACs, premature atrial contractions; PVCs, premature ventricular contractions; SB, sinus bradycardia; CDs, conduction disorders; WOAs, without arrhythmias.

The median follow-up duration was 18.3 (9.9–29.2) months. There was no significant difference in all-cause mortality between the group with arrhythmias and the group without arrhythmias (*P* > 0.05). The prognostic value of different types of arrhythmias was also evaluated. The all-cause mortality rates of patients with ST, SB, AF, PACs, PVCs, and CDs and in patients without arrhythmias were 60.4% (29/48), 34.8% (16/46), 73.7% (14/19), 60.0% (12/20), 28.6% (4/14), 52.6% (10/19), and 50.6% (84/166), respectively (Figure 2). The patients with SB had lower all-cause mortality than those with AF (*P* < 0.01).

Furthermore, in the subgroup analysis of patients with different arrhythmias, univariate Cox analysis showed that there was a significant positive correlation between SB and survival [hazard ratio (HR): 0.500 [0.298–0.837], *P* = 0.008]. In addition, SB was also independently negatively associated with all-cause mortality (HR: 0.592 [0.352–0.998], *P* = 0.049) (Table 3).

**Table 3:** Univariate and multivariate Cox regression analysis for all-cause mortality.

	**Univariate analysis**			**Multivariate analysis**		
	**HR**	**95% CI**	***P***	**HR**	**95% CI**	***P***
Age (years)	1.008	0.992–1.023	0.327			
Male	1.275	0.929–1.752	0.133			
Log NT-proBNP (pg/mL)	1.914	1.565–2.341	<0.0001	1.627	1.312–2.018	<0.0001
LDH (U/L)	1.002	1.001–1.002	<0.0001	1.002	1.001–1.002	<0.0001
eGFR (mL/min/1.73 m^2^)	0.992	0.989–0.996	<0.0001	1.000	0.995–1.004	0.853
Serum albumin (g/L)	0.986	0.963–1.010	0.253			
Serum potassium (mmol/L)	0.971	0.729–1.295	0.843			
Hemoglobin (g/L)	0.985	0.978–0.992	<0.0001	0.993	0.985–1.001	0.105
CAD	1.724	0.995–2.989	0.052			
Hypertension	1.099	0.791–1.527	0.575			
Diabetes	1.521	0.986–2.347	0.058			
**ISS Stage**, ***n*** **(%)**						
I	1			1		
II	1.746	0.996–3.062	0.052	1.425	0.794–2.555	0.235
III	3.196	1.837–5.563	<0.0001	2.004	1.051–3.824	0.035
AF	1.664	0.961–2.880	0.069			
PACs	1.705	0.945–3.076	0.076			
PVCs	0.494	0.183–1.335	0.164			
ST	1.485	0.992–2.223	0.054			
SB	0.500	0.298–0.837	0.008	0.592	0.352–0.998	0.049
CDs	1.099	0.580–2.086	0.772			
**Types of arrhythmias**						
0	1					
1	1.138	0.834–1.555	0.415			
>1	0.943	0.411–2.163	0.890			

*HR, hazard ratio; NT-proBNP, N-terminal pro-brain natriuretic peptide; LDH, lactate dehydrogenase; eGFR, estimated glomerular filtration rate; CAD, coronary artery disease; ISS, International Staging System; AF, atrial fibrillation; PACs, premature atrial contractions; PVCs, premature ventricular contractions; ST, sinus tachycardia; SB, sinus bradycardia; CDs, conduction disorders*.

## Discussion

Approximately half of the patients (48.0%) had arrhythmias in our study, including ST (15.0%), SB (14.4%), PACs (6.3%), CDs (6.0%), AF (6.0%), PVCs (4.4%), and PSVT (0.6%). Patients with SB had lower all-cause mortality than those with AF, which might be an independent positive factor for prognosis.

Advances in the treatment and management of MM patients have significantly prolonged survival; however, the number of patients with cardiovascular complications is increasing. In previous studies, arrhythmias have been shown to be common in MM patients and to cause undesirable results. In a retrospective cohort study of 32,193 patients, nearly 60% of patients experienced cardiac events. The incidence of cardiac arrhythmias was 14% for newly diagnosed patients and 17% for relapsed patients, and the incidence of CDs was 2% for newly diagnosed and 2% for relapsed patients (21). In a large national database of 88,507 patients, 20% were found to have arrhythmias, with the most common type being AF (67.7%), followed by paroxysmal ventricular tachycardia (5.0%), atrial flutter (3.9%), and paroxysmal atrial tachycardia (2.2%) (6). The incidence of arrhythmias was high in our study, with ST (15.0%) and SB (14.4%) occurring more frequently than AF (6.0%). Many factors, including comorbidities, cardiac function, organ dysfunction and various cardiotoxic chemotherapeutics, can increase the incidence of arrhythmia.

The distribution of diabetes was found to be similar between patients with and without arrhythmias in the MM population in a previous study (6); however, there is increasing evidence that the presence of diabetes impacts the incidence of arrhythmia. Diabetes is closely associated with cardiac arrhythmias, especially in patients with chronic kidney disease, hypoglycemia and hyperglycemia, and the most common arrhythmias are AF, conduction abnormalities, and ventricular arrhythmias (22–26). Patients with MM often experience renal dysfunction, electrolyte abnormalities, anemia and hyperglycemia, which are factors for arrhythmia. The percentage of patients with diabetes was higher in the group with arrhythmias than in the group without arrhythmias, and diabetes might aggravate the risk of cardiac arrhythmias among MM patients in our study.

Left ventricular ejection fraction was evaluated and compared, but no significant differences were observed between the group with arrhythmias and the group without arrhythmias. NT-proBNP is another important measure of cardiac function; with high levels of NT-proBNP, cardiac function may be affected (27), and heart failure can even develop. Given the retrospective nature of our study, the incidence of the exact type of heart failure could not be determined, but NT-proBNP was found to be an independent predictor of arrhythmias after adjusting for creatinine.

The rate of arrhythmias in relapsed MM patients was higher than that in newly diagnosed patients (21). Although a large difference was observed between relapsed patients and newly diagnosed patients, to some extent, this finding indicated that the tumor burden in MM patients might increase the incidence of arrhythmias. In our study, tumor burden was assessed by ISS stage and specific clinical features, such as hypercalcemia, renal insufficiency, and anemia. Renal damage has been linked to higher rates of arrhythmias and sudden death (28), and anemia has been shown to increase the risk of arrhythmias and hypertension (29, 30). However, there was no difference in these indicators between the group with arrhythmias and the group without arrhythmias in our study.

Arrhythmias were also the most common cardiovascular complications during anti-myeloma therapy treatment with proteasome inhibitors, immunomodulators, corticosteroids, alkylating agents, anthracyclines, etc. (15). A large retrospective study reported a low incidence of cardiac arrhythmias with bortezomib-based treatment (31). In our study, approximately half of the patients received bortezomib and the proportion of patients who received bortezomib (54.2 vs. 40.4%, *P* = 0.013) was higher in patients with arrhythmias than in those without arrhythmias for the following reasons. First, prescription bortezomib is the most recommended medication, especially among patients with cardiovascular risk factors or comorbidities. Second, lenalidomide use is limited in patients with renal dysfunction and is cost prohibitive. Third, carfilzomib is not easy to obtain. Based on the above factors, a high rate of bortezomib use was accepted in the arrhythmia group.

The distribution and incidence rates of SB were 30.1% (46/153) and 14.4% (46/319), respectively. The rates of patients who received thalidomide, cyclophosphamide and anthracycline were 22.3, 42.3, and 42.0%, respectively. Chemotherapy agents associated with bradycardia in patients with MM have been described in previous studies. Thalidomide and lenalidomide have been associated with the development of bradyarrhythmias and AF, and the incidence of SB in patients is 1–10% (10, 32). Other studies showed that SB was observed in ~26–53% of patients who received thalidomide as initial therapy for early-stage myeloma (33) and was found in more than 10% of patients who received cyclophosphamide (34). Apart from thalidomide- or lenalidomide-induced arrhythmias, most anthracycline-induced arrhythmias are benign; however, critical bradycardia and AF have been reported (35, 36). Limited by the small cohort and the single-center retrospective design, it was difficult to identify the relationship between chemotherapy agents and different types of arrhythmias.

Cardiac amyloidosis might lead to bradycardia, low voltage and atrioventricular block, and these novel agents and their neurotoxicity exacerbate bradycardia (37). Although endocardial biopsy and speckle tracking echocardiography technology are conducive to the early detection and diagnosis of cardiac amyloidosis, they are not widely used at our center, and the proportion of patients with cardiac amyloidosis is unclear.

Sex differences are also an important factor and should be considered due to their impact on diagnosis, cardiovascular complications, and therapeutic interventions. Different levels of hormones may affect the incidence and prevalence of arrhythmias between men and women. Little is known about sex differences in arrhythmias among MM populations. In this study, the incidence rates of arrhythmias in women and men were 17.2 and 30.7%, and no significant difference was found between women and men (*P* > 0.05). For example, the percentage of AF in women was higher than that in men, and the all-cause morbidity of AF in women was lower than that in men. These findings are similar to the previous studies (38, 39); however, no significant differences in the incidence of AF and all-cause morbidity between males and females were found in our study, which may be due to the small size of cases and other factors.

SB was found to be a positive prognostic factor for survival in MM patients in this study; this finding was consistent with the results of previous studies (40–42). One retrospective cohort study indicated that bradycardia was associated with good neurologic outcomes during therapeutic hypothermia for comatose survivors of out-of-hospital cardiac arrest and should not be aggressively treated during this period (40). Patients with relative bradycardia were found to have lower mortality, even after adjusting for the confounding factor of septic shock disease (41). Similarly, Kyriazopoulou et al. noted that cardiac arrest patients who exhibited SB during targeted temperature management had a lower 180-day mortality and better final neurological prognosis (42). However, it is challenging to determine the value of bradycardia. Moreover, bradycardia was not associated with incident cardiovascular disease or mortality in a community-based cohort (43). Bradycardia is usually considered an adverse prognostic indicator; severe or prolonged bradycardia might cause heart failure, hypotension, and syncope. The prognosis of patients with symptomatic bradycardia was found to be poor if they were not treated with a pacemaker. Temporary bradycardia in patients with COVID-19 was determined to be associated with a high rate of short-term morbidity and poor outcomes (44). The common causes of bradycardia are structural heart disease, CDs, or other cardiac conditions. In terms of pathology, dysfunction of the sinus node, atrioventricular nodal tissue, and the specialized His-Purkinje conduction system might predispose patients to bradycardia. SB was a main kind of bradycardia in our study, with a percentage of 97.8% (45/46), and only one patient had conduction disorder. The positive effects of SB on mortality might be because a lower heart rate could reflect depressed sympathetic activation. However, the exact mechanisms underlying this relationship await further study.

The present study had several potential limitations. First, this was a study with small size. Second, our study had a single-center retrospective design; therefore, clinical data related to the treatment of coexisting cardiac disease, antiarrhythmic drugs, dynamic ECG, the atropine test and patient response, chemotherapeutic regimens, and the out-of-hospital use of thalidomide were not available. Third, speckle tracking echocardiography technology and imaging technology were not widely used in our center, so the proportion of patients with cardiac amyloidosis was unclear.

## Conclusions

Patients with MM had a heavy arrhythmia burden, and in this study, approximately half of MM patients had arrhythmias. MM patients with SB were associated with a lower all-cause mortality rate than those with AF. SB might be an independent positive factor for prognosis. Arrhythmias should be considered in all patients and could help to guide cardiovascular risk assessment.

## Data Availability Statement

The raw data supporting the conclusions of this article will be made available by the authors, without undue reservation.

## Ethics Statement

The studies involving human participants were reviewed and approved by the Ethics Committee of the First Affiliated Hospital of Xi'an Jiao Tong University (Approval No. XJTU1AF2020LSK-179). The patients/participants provided their written informed consent to participate in this study.

## Author Contributions

CS and JW contributed to the conception and design of the work and critically revised the manuscript. MT critically revised the manuscript. MT, LZ, SW, JS, and HL contributed to the acquisition, analysis, and interpretation of data. YL drafted the manuscript. All authors approved the manuscript and agreed to be accountable for all aspects of the work, ensuring its integrity and accuracy.

## Funding

The study was funded by the Key Research and Development Program of Shaanxi (No. 2020SF-171) and the Institutional Foundation of The First Affiliated Hospital of Xi'an Jiaotong University (No. 2020ZYTS-20, 2018QN-05).

## Conflict of Interest

The authors declare that the research was conducted in the absence of any commercial or financial relationships that could be construed as a potential conflict of interest.

## Publisher's Note

All claims expressed in this article are solely those of the authors and do not necessarily represent those of their affiliated organizations, or those of the publisher, the editors and the reviewers. Any product that may be evaluated in this article, or claim that may be made by its manufacturer, is not guaranteed or endorsed by the publisher.
