# Supplementary material for: Incidence of Arrhythmias and Their Prognostic Value in Patients With Multiple Myeloma
Source: Front Cardiovasc Med. 2021 Nov 15;8:753918. doi: 10.3389/fcvm.2021.753918 (PMC8634844; doi:10.3389/fcvm.2021.753918)
Supplement: Supplementary file 1 [file Data_Sheet_1.docx]

Supplementary Table 1. Logistic regression analysis for arrhythmias.

|  | **HR** | **95%CI** | ***P* value** |
| --- | --- | --- | --- |
| Log NT-proBNP (pg/mL) | 1.413 | 1.032-1.934 | 0.031 |
| Creatinine (mg/dL) | 1.030 | 0.923-1.150 | 0.595 |
| Bortezomib | 1.599 | 1.002-2.552 | 0.049 |
| Supportive care | 0.439 | 0.189-1.020 | 0.056 |
| Diabetes | 2.102 | 1.026-4.304 | 0.042 |
| HR, hazard ratio; NT-proBNP, N-terminal pro-brain natriuretic peptide. | | | |

Supplementary Table 2. The details of patients with more than one type of arrhythmia.

| **Types of arrhythmias** | **Number of patients** |
| --- | --- |
| ST, RBBB and LBBB | 1 |
| LBBB and PACs | 1 |
| RBBB and AF | 1 |
| RBBB and PACs | 1 |
| RBBB and PVCs | 1 |
| RBBB and ST | 3 |
| SB and first-degree AV block | 1 |
| PSVT and Mobitz I AV block | 1 |
| PVCs and PACs | 1 |
| PVCs and AF | 1 |
| PVCs and SB | 2 |
| PACs and SB | 1 |
| **Total** | 15 |
| ST, Sinus tachycardia; RBBB, Right bundle branch block; LBBB, Left bundle branch block; PACs, Premature atrial contractions; AF, Atrial fibrillation; PVCs, Premature ventricular contractions; SB, Sinus bradycardia; AV, Atrioventricular. | |
